# Teaching in Hospital Educational Centers: Navigating Educational Dialogue in Multifaceted Context

**DOI:** 10.5334/cie.268

**Published:** 2026-04-15

**Authors:** Meirav Hen, Adi Hadari, Arie Kizel

**Affiliations:** 1 Tel Hai University of Kiryat Shmona in the Galilee, IL; 2 Haifa University, IL

**Keywords:** hospital pedagogy, student–patient conflict, pediatric oncology, qualitative research, pedagogical respite, student autonomy

## Abstract

Hospital teachers in pediatric oncology departments operate at the intersection of medical and educational institutions, navigating a complex environment in which the need for clinical care often subordinates pedagogical goals. This study explores the professional experiences of these educators as they manage the inherent tension between the “logic of schooling” and the “logic of medicine”. Using a qualitative-constructivist framework, the study conducted semi-structured interviews with 13 expert teachers and one national supervisor across four diverse Israeli medical centers. Data was analyzed using thematic analysis to identify the core strategies used to bridge competing institutional demands. The analysis identifies the “student–patient conflict” as the defining structural tension of hospital pedagogy, arising from the child’s dual role as a learner and a patient. To resolve this conflict, teachers employ three primary strategies: Positioning learning as a voluntary choice to restore the agency and control lost in the patient role, helping students master their clinical environment as a form of cognitive empowerment, and using play, humor, and spatial shifts to move children out of “sick spaces,” thereby reclaiming the child’s “healthy self” and student identity. The findings suggest that hospital pedagogy in oncology is defined less by academic instruction and more by the reclamation of identity. The study highlights a significant gap between standardized Ministry of Education policies and the fragmented reality of the ward. It recommends an institutional shift toward recognizing “pedagogical respite” as a valid educational outcome, prioritizing flexibility and emotional resilience over traditional curricula to better support students in crisis.

Hospital teachers, particularly those working within pediatric oncology departments, occupy a unique and emotionally charged position at the intersection of two powerful institutional systems (Kazak et al., 2018). Unlike traditional educational settings, hospital-based education is characterized by a fundamental duality: the simultaneous necessity of medical treatment and the preservation of educational continuity (Capurso & Dennis, 2017). In this environment, the pedagogical role is frequently subordinated to medical priorities, creating a landscape where the “student” identity must compete with the “patient” identity for space and attention (Hen & Gilan Shochat, 2022).

The physical and temporal boundaries of the hospital classroom further reflect this tension. Unlike the traditional school setting defined by fixed timetables and dedicated spaces, hospital education occurs in “fluid” spaces at the bedside or within treatment areas, dictated entirely by the rhythm of medical procedures (Ludgerio et al., 2023). This unpredictable environment requires teachers to operate in a state of constant adaptation, often without prior knowledge of their students’ daily health status or availability (Äärelä et al., 2016). This unpredictability is not merely a logistical hurdle; it represents a primary friction between the structured expectations of schooling and the erratic nature of medical crisis.

Furthermore, the role of the hospital teacher extends far beyond academic instruction into psychosocial mediation. In Western contexts such as the United States and the United Kingdom, specialized roles, such as Child Life Specialists or Playleaders, support the child’s emotional and developmental coping (Romito et al., 2021). However, in the Israeli context, hospital teachers often absorb these responsibilities, acting as the primary mediators between the child’s world and the clinical environment (Pinchover, 2019). Studies indicate that direct instruction accounts for less than half of a teacher’s working hours, with much of their time dedicated to navigating complex collaborations with medical teams, parents, and community schools (Steinke et al., 2016).

This expansion of the teacher’s role into mediation and “respite” (Hopkins, 2016) suggests that the teacher is not merely an educator, but a negotiator of the child’s institutional status. Whether facilitating a student’s reintegration into their “healthy” community school or using games to provide an emotional break from treatment, the teacher is constantly bridging the gap between the world of the “well” and the world of the “sick” (Csinády, 2015).

Building on these complexities, this study utilizes a qualitative research framework to explore the “student–patient conflict”. We conceptualize this conflict as the persistent structural and experiential tension teachers face when children simultaneously assume the roles of learners and patients. Rather than focusing solely on pedagogical techniques or care ethics, this study highlights the structural incompatibility between the “logic of schooling” defined by continuity, curricular progression, and assessment, and the “logic of medical care” defined by vulnerability, uncertainty, and treatment-driven schedules. Ultimately, we aim to foreground how teachers actively negotiate these institutional contradictions within their daily pedagogical practice.

## Methods

### Research Design

This study employed a qualitative-constructivist methodology (Shkedi, 2007) to explore the nuanced experiences and professional expertise of hospital teachers. This approach was chosen to capture educators’ subjective perspectives as they navigate the “student–patient conflict,” the structural tension between pedagogical objectives and the medical realities of pediatric oncology. By utilizing a constructivist lens, the study foregrounds the meanings teachers ascribe to their daily practices and the strategies they develop to bridge competing institutional logics.

### Participants

The research sample comprised 13 teachers from four distinct hospital educational centers in Israel, supplemented by a national supervisor from the Ministry of Education. The selection of these centers followed a purposive sampling strategy to ensure maximum variation across geographic and organizational contexts: two oncology departments within large, central general medical centers. One specialized pediatric department within a major children’s hospital. And one oncology unit within a peripheral hospital in the northern region. In all settings, instruction was characterized by high levels of individualization, delivered primarily via individual teaching or in small-group clusters.

The study’s sample comprises a highly experienced (average of 23.5 years of total teaching experience), academically qualified group of professionals within the Israeli hospital education system (61% of the teachers hold a master’s degree). Furthermore, many participants have undergone “conversion” training in Special Education. In addition, the sample encompasses a broad spectrum of educational roles, including early childhood/kindergarten teachers (23%), subject-specific instructors (e.g., English, Hebrew, Mathematics), and arts-based educators (see Table 1 in the Appendix).

Participants were recruited through the principals of each educational center, who identified the most experienced teachers for potential inclusion. Of the 15 teachers approached, 13 consented to participate (87% response rate). All participants were recognized as experts in hospital pedagogy. To ensure ethical rigor, the study received formal approval from the University of Haifa Ethics Committee (2021). All participants signed informed consent forms, and confidentiality was maintained using pseudonyms and Latin letter codes for the hospitals.

### Data Collection

Primary data were gathered through semi-structured interviews conducted by the first author between 2021 and 2022. The interviews, averaging 60 minutes, were designed to elicit thick descriptions of the teachers’ daily practices, professional challenges, and coping mechanisms within the oncology ward. To enhance ecological validity, most interviews were conducted in person within the hospital. All sessions were audio-recorded and transcribed verbatim. The researcher maintained a reflexive journal throughout the process to account for the potential influence of their role as an MA student on the interview dynamics, ensuring a supportive and non-judgmental atmosphere.

### Data Analysis

The data were analyzed using thematic analysis in three recursive phases. While this analysis draws on a broader research project (Hadari et al., 2025), it was explicitly conducted to examine the educational dynamics unique to pediatric oncology.

#### Phase 1: Immersion and Initial Categorization

The research team engaged in multiple readings of the transcripts to achieve data immersion. The first author performed initial coding, which the two co-authors subsequently audited to identify recurring patterns. Dedoose software was utilized to classify the data into meaningful units while preserving contextual integrity (Shkedi, 2007).

#### Phase 2: Systematic Mapping

The researchers collaboratively mapped the initial codes to characterize the core elements of the teachers’ roles, focusing on the specific challenges and educational philosophies that emerge at the intersection of school and medicine.

#### Phase 3: Identification of the Core Category

In the final phase, the team identified the “student–patient conflict” as the core category. This central theme was further interpreted to reveal the nuances of how teachers negotiate the tension between educational continuity and the “sick space” of the hospital.

## Results

The analysis of the teachers’ narratives revealed several core categories that reflect the complexities of hospital-based teaching in pediatric oncology departments. These categories highlight the dynamic balance between educational objectives and the unique challenges of working with hospitalized students. The analysis reveals that hospital-based teaching in pediatric oncology is characterized by a basic “Student–Patient Conflict”. This conflict arises from the structural friction between the “logic of schooling” (continuity, goals, and assessment) and the “logic of medicine” (vulnerability, treatment schedules, and physical crisis) (See Table 2).

**Table 2:** Core Categories and Key Themes.


CORE CATEGORY	KEY THEME	EXEMPLARY QUOTE/EXAMPLE

**Autonomy & Agency**	Education as a Voluntary Choice	*“The children’s participation… is entirely voluntary… that is their full right.”* (Lotem)

**The Dialogue of Negotiation**	Respecting Boundaries & Space	Teachers knocking and asking permission to enter the room to restore a sense of control.

**Fragmented Continuity:**	Reaching Out via Shared Activities	Joining a student in *Fortnite* or phone games to build a bridge between teacher and student.

	Fragmented Routine & Continuity	*“Learning is always done in a way that it can be interrupted—resumed after half an hour or two weeks.”*

	Healthy Self vs. Sick Space	Using “Community School” links or moving the child out of pajamas/bed to reconnect with their “student” identity.

**Institutional Gap**	Policy Gap (Ministry vs. Reality)	The clash between standardized Ministry plans and the actual “different” work of hospital pedagogy.


### 1. Autonomy and Agency

Teachers conceptualize Education as a choice to mitigate the loss of control inherent in being a patient. Because medical treatment is mandatory and often invasive, teachers position learning as the only voluntary domain. By making participation entirely voluntary, teachers use the “student” role to restore the agency that the “patient” role strips away.

As *Iris* (T6) explained, “My goal is for the child who comes to our department to receive treatment. First and foremost, they come here for treatment, not for me or to learn with me.” Similarly, *Lotem* (T10) remarked, “The children’s participation in learning processes is entirely voluntary, according to their wishes and preferences… There are many cases where children do not want to participate, and that is their full right.”

*Rakefet* (T1) explained, “As much as possible, I want them to feel in control. Hospitalization inherently involves a loss of control and the ability to rebel.” *Rakefet* described her work with a student who had stopped reading due to illness to reconnect with a previous interest. “My role is to read with her, to bring back something from her old self, the ability to immerse herself in her imagination.”

### 2. The Dialogue of Negotiation

The conflict is physically manifested in the hospital room. Teachers practice a specific “dialogue of space,” such as knocking and waiting for permission to enter, to respect the student’s emotional and physical boundaries. This is a direct negotiation of the conflict: the teacher (representing the school) must ask for entry into a space governed by medical necessity. Flexibility becomes the primary tool for resolving this tension, especially when medical side effects, such as steroid-induced mood swings, clash with pedagogical goals.

*Rakefet* (T1) described her approach: “First, I knock, and then after a few seconds, I open the door and say, ‘Good morning,’ addressing the child by name. I never enter immediately. When they respond, I ask, ‘May I come in?’” Likewise, *Lilach* (T7) shared, “I always knock first; I make an effort not to enter right away.”

*Havazzelet* (T4) shared, “Some kids are glued to their phones, so I say, ‘Teach me what you are doing.” Similarly, *Vered* (T8) recounted, “A child was playing Fortnite, and I said, ‘Teach me how to play.’ I spent hours with him.” By adapting to students’ interests, teachers created opportunities for engagement that transcended traditional educational content.

### 3. Fragmented Continuity: The Clash of Schedules

The results highlight a “fragmented educational work” reality. The logic of schooling requires routine, yet the logic of medicine is driven by the “fluidity of time” and sudden interruptions. Teachers attempt to “preserve the flow” and create “mutual responsibility,” yet they must constantly yield to medical calls. This results in an educational experience that is “set aside and resumed,” moving between the child’s identity as a learner reaching for future milestones (like matriculation exams) and a patient focused on immediate survival.

As *Shoshana* (T3) described, “The lesson depends on the child. Sometimes it’s 45 minutes, sometimes just 15 minutes because they get called for treatment. Everything is very flexible.” *Lotem* (T10) noted, “Learning is always done in a way that can be interrupted, set aside, resumed after half an hour, two weeks, or even months.” *Rakefet* (T1) highlighted the importance of consistency: “The goal is to preserve the flow… at least one lesson per week, to create some mutual responsibility.”

### 4. The Institutional Gap: Policy vs. Bedside Reality

A final layer of the student–patient conflict exists at the policy level. Teachers reported a significant gap between the Ministry of Education’s standardized expectations and the realities on the ground. While the Ministry demands structured curricula, the reality of oncology requires a “dialogue through games” and “facilitating joy”, methods that prioritize the child’s emotional resilience over formal academic output.

*As Narkis (T9) pointed out, “The Ministry demands plans and activities very similar to those in regular schools and kindergartens. They do not fully understand how different this work is.” Lotem* (T10) shared, “It is appropriate to give the child space to express themselves, and I should respond and guide them.” At the same time, facilitating joy was a key goal. *Nurit* (T2) stated, “My goal is to get them out of bed smiling. That is my aim, to make them happy… I do not understand anything about their recovery, but I believe it hurts less if I laugh more.” As *Vered (T8) noted*, “Learning can come from anything, even from a game. Moreover, even if you sit down to play sometimes and there is nothing there, a connection will still be made.

## Discussion

The findings of this study illuminate the “Student–Patient Conflict” not merely as a challenge, but as the defining structural tension of hospital pedagogy. While previous research often emphasizes the “pedagogy of care” (Aujoulat et al., 2006) or developmental support (Romito et al., 2021), our results suggest that the teacher’s role in pediatric oncology is a constant negotiation between the logic of schooling (continuity, assessment, and identity) and the logic of medicine (vulnerability, urgency, and institutional power).

### The Student–Patient Conflict as a Structural Contradiction

At the heart of our findings is the conflict between the child’s dual roles. As a patient, the child is subject to the medical logic of compliance and bodily treatment; as a student, they are expected to engage in growth, inquiry, and future-oriented goals. This study moves beyond existing frameworks by foregrounding how this contradiction is evident in every pedagogical interaction, from knocking on a hospital door to modifying curricula to fit a “treatment-driven schedule.”

### Negotiated Autonomy and the Curriculum of Choice

While Western contexts often prioritize curriculum alignment with community schools to ensure continuity (Steinke et al., 2016), Israeli hospital teachers resolve the student–patient conflict by prioritizing absolute student autonomy (Hen, 2020). By allowing learners to choose the subject, timing, and depth of their studies, teachers transform the “student” role into a sanctuary of agency. As Biesta (2013) notes, the teacher’s role is to introduce new elements into the child’s life; in the oncology ward, the “new element” is the right to say “no.” This collaborative determination of the curriculum serves as a counter-logic to the medical environment, where the child often has little choice regarding their treatment. This approach aligns with the “pedagogy of searching” (Kizel, 2024), where learning becomes a present-focused tool for emotional resilience rather than just a race for academic credits.

### Mediation as a Bridge Between Logics

A key finding is the teacher’s role in mediating the medical environment. Unlike Child Life Specialists, who focus on developmental liaison (Romito et al., 2021), teachers in this study perceive medical mediation as an educational act. By helping students understand and “master” their procedures, teachers help the child reclaim a sense of control over their “patient” identity. This mediation reduces the friction of the student–patient conflict by integrating the medical reality into the learning process, thereby transforming a distressing medical event into a moment of cognitive and emotional mastery.

### Pedagogical Respite and the Reclamation of the “Healthy Self”

Our findings introduce the concept of Pedagogical Respite as a vital resolution to the institutional tension. Teachers utilize games, humor, and crafts not merely as “time-fillers,” but as a deliberate strategy to suspend the patient’s identity. Unlike play therapy models that reflect developmental needs (Cameron & Patte, 2018), teachers use games to build a “connection” that is intentionally unrelated to illness. In addition, by encouraging students to leave their “sick space” (beds and pajamas), teachers facilitate a physical transition from “patient” back to “student.” This respite allows for what we term the “reclamation of the healthy self,” providing a temporary exit from the medical logic and a return to a world defined by joy and “authentic dialogue.”

### Institutional Tension: Policy vs. Ward Reality

Finally, the study highlights a discrepancy between the Ministry of Education’s standardized expectations and the ward’s reality. This “policy conflict” forces teachers to play a dual role: they must satisfy the Ministry’s institutional logic while practicing a highly individualized, flexible pedagogy that prioritizes the child’s immediate vulnerability. This underscores the need for policy frameworks that recognize the unique “fragmented” nature of hospital teaching, moving away from rigid matriculation goals toward a more holistic, adaptive educational model.

### Limitations

While this study provides deep insights into the experiences of hospital teachers in Israel, several limitations should be noted:

***Contextual Specificity:*** The study focused on teachers within pediatric oncology departments in Israel. Given the unique cultural and institutional structure of the Israeli educational and medical systems, the findings may not be directly transferable to other countries or different medical specialties (e.g., psychiatry or rehabilitation).

***Methodological Scope:*** As a qualitative study involving 13 teachers and one national supervisor, the findings aim for analytical depth rather than statistical generalizability. The small sample size reflects a specific group of “expert” teachers, whose experiences may differ from those newer to the field.

***Perspective Bias:*** The data relies on the self-reported narratives of teachers. Future research would benefit from incorporating the perspectives of hospitalized children themselves, their parents, and medical staff to provide a multi-vocal understanding of the student–patient conflict.

### Practical Implications

The findings offer several actionable recommendations for policymakers, hospital administrators, and educators. It suggests that the Ministry of Education should develop specialized assessment tools and curricula that acknowledge the “fragmented” nature of hospital teaching. Recognizing “Pedagogical Respite” and emotional support as valid educational outcomes, rather than focusing solely on matriculation, would reduce the institutional pressure on teachers. Also, professional development for hospital teachers should include training in medical mediation and “pedagogical negotiation.” Understanding the logic of the medical ward can help teachers navigate the student–patient conflict more effectively. In addition, hospitals should foster formal “dialogue spaces” where teachers and medical staff can align their goals. Recognizing the teacher as a “reclaimer of the healthy self” can help medical staff see education not as an interruption, but as a vital component of the child’s holistic recovery. Finally, hospital departments should prioritize creating “non-medical” learning spaces (classrooms or workshops) that allow children to physically leave their “sick space” and enter a “student space,” reinforcing their healthy identity.

### Conclusion

This study conceptualizes the work of hospital teachers through the lens of the student–patient conflict, a structural tension between the demands of schooling and the unpredictable realities of medical needs. Our findings demonstrate that hospital teachers in pediatric oncology do not merely “teach subjects”; they act as expert negotiators who bridge two competing institutional worlds. Through the practice of Pedagogical Respite, teachers use choice, play, and authentic dialogue to help children reclaim their “healthy self” and maintain a sense of agency in an environment that often strips it away. By moving beyond traditional pedagogical frameworks, this research highlights that in the hospital setting, the most profound educational act is often the restoration of the child’s identity as a learner. Ultimately, supporting these teachers requires an institutional shift that values flexibility over standardization and recognizes that for a hospitalized child, learning is a powerful form of resilience.

## Appendix

**Table 1:** Demographic Data of Research Participants.

HOSPITAL CODE	PARTICIPANT NO.	TOTAL TEACHING EXP. (YEARS)	DEPT. EXP. (YEARS)	PROFESSIONAL ROLE	ACADEMIC BACKGROUND

**Hospital A**	T1	21	6	Art Teacher (Jewelry)	B.A. Art & Teaching Cert.

	T2	20	7	English Teacher	B.A. English & Bibliotherapy

	T4	40	18 (5 in Dept.)	Educator & Floor Manager	M.A. Ed. Administration

**Hospital B**	T9	14	14 (3 in Dept.)	Kindergarten Teacher	B.Ed. Special Education

	T10	42	4	Educator	M.A. Bible Studies & Special Ed.

	T13	25	8 (4 in Dept.)	Teacher	B.A. Special Education

**Hospital C**	T6	5	5	Art Teacher	B.A. Art & Teaching Cert.

	T7	30+	30+	Lead Teacher & Floor Manager	M.A. Digital Communication & Special Ed.

	T8	19	5	Lead Teacher	M.A. Educational Counseling

	T11	—	3	Psychology Instructor	M.A. Candidate (Psychology)

**Hospital D**	T3	18	12	Kindergarten Teacher	B.Ed. Special Education

	T5	9	7	Hebrew & Language Teacher	M.A. Learning Disabilities

	T12	30	27	Elementary Educator	B.Ed. Early Childhood

**National**	Supervisor	35	15	National Supervisor	M.A. Occupational Therapy & Counseling

## References

[1] Äärelä, T., Määttä, K., & Uusiautti, S. (2016). Ten encounters between students and a special education teacher at a Finnish hospital school – Outlining hospital school pedagogy. Global Journal of Human-Social Science: Linguistics and Education, 16(4), 9–20.

[2] Aujoulat, I., Simonelli, F., & Deccache, A. (2006). Health promotion needs of children and adolescents in hospitals: A review. Patient education and counseling, 61(1), 23–32. 10.1016/j.pec.2005.01.015 16533675

[3] Biesta, G. J. (2013). The beautiful risk of education. Routledge.

[4] Cameron, C. B., & Patte, M. M. (2018). A Typology of Play in Medical Settings. In P. Smith, & J. Roopnarine (Eds.), The Cambridge Handbook of Play: Developmental and Disciplinary Perspectives (615–629). New York: Cambridge University Press.

[5] Capurso, M., & Dennis, J. L. (2017). Key educational factors in the education of students with a medical condition. Support for Learning, 32(2), 158–179. 10.1111/1467-9604.12156

[6] Csinády, R. (2015). Hospital pedagogy: A bridge between hospital and school. Hungarian Educational Research Journal, 5(2), 49–65.

[7] Hadari, A., Hen, M., & Kizel, A. (2025). The work of teachers in the hospital education center: Educational dialogue in multiple arenas. Studies in Education: Journal for Study and Research in Education (Iyunim BaHinuch), 25, 224–23. (Hebrew).

[8] Hen, M. (2020). Teaching Emotional intelligence: An academic course for hospital teachers. Continuity in Education, 1(1), 22–36. 10.5334/cie.13 38774526 PMC11104398

[9] Hen, M. & Gilan-Shochat, M. (2022). Exploring the unique professional identity of hospital teachers in Israel. Continuity in Education, 3(1), 115–126. 10.5334/cie.46 38774286 PMC11104386

[10] Hopkins, L. J. (2016). Hospital-based education support for students with chronic health conditions. Australian Health Review, 40(2), 213–218. 10.1071/AH15032 26412444

[11] Kazak, A. E., Scialla, M. A., Patenaude, A. F., Canter, K., Muriel, A. C., Kupst, M. J., Chen, F. F., & Wiener, L. (2018). The multidisciplinary pediatric psycho-oncology workforce: A national report on supervision for staff and training opportunities. Journal of Pediatric Psychology, 43(10), 1101–1109. 10.1093/jpepsy/jsy047 PMC6715455 30242934

[12] Kizel, A. (2024). Enabling students’ voices and identities: philosophical inquiry in a time of discord. Lexington Books. 10.5040/9798216432739

[13] Ludgerio, M. J. B., Pontes, C. M., Dos Santos, B. L. C., Macedo, E. C., Marinus, M. W. D. L. C., & Leal, L. P. (2023). Pedagogical practices developed with children through hospital classes: An integrative literature review. Journal of Pediatric Nursing, 72, e10–e18. 10.1016/j.pedn.2023.05.014 37302968

[14] Pinchover, A. (2019). School for hospitalized children: Vision and implementation at the Hadassah Medical Center. Dvarim, 12, 75–95. [Hebrew]

[15] Romito, B., Jewell, J., & Jackson, M. (2021). Child life services. Pediatrics, 147(1), e2020040261. 10.1542/peds.2020-040261 33372119

[16] Shkedi, A. (2007). Words that attempt to touch: Qualitative research – Theory and practice. Ramot. [Hebrew]

[17] Steinke, S. M., Elam, M., Irwin, M. K., Sexton, K., & McGraw, A. (2016). Pediatric hospital school programming: An examination of educational services for students who are hospitalized. Physical Disabilities: Education and Related Services, 35(1), 28–45. 10.14434/pders.v35i1.20896

